# Characterization of Plant Homeodomain Transcription Factor Genes Involved in Flower Development and Multiple Abiotic Stress Response in Pepper

**DOI:** 10.3390/genes14091737

**Published:** 2023-08-30

**Authors:** Jinfen Wen, Minghua Deng, Kai Zhao, Huidan Zhou, Rui Wu, Mengjuan Li, Hong Cheng, Pingping Li, Ruihao Zhang, Junheng Lv

**Affiliations:** 1 Faculty of Architecture and City Planning, Kunming University of Science and Technology, Kunming 650500, China; wenjf888@163.com; 2 College of Landscape and Horticulture, Yunnan Agricultural University, Kunming 650201, China; dengminghua2013@sina.com (M.D.); kailixian1023@aliyun.com (K.Z.); zhd1025@163.com (H.Z.); wr1236542020@163.com (R.W.); 13150515440@163.com (M.L.); chengh0222@163.com (H.C.); liping-1200@163.com (P.L.); zrh@yaas.org.cn (R.Z.)

**Keywords:** pepper, PHD, flower development, abiotic stress, hormonal response

## Abstract

Plant homeodomain (PHD) transcription factor genes are involved in plant development and in a plant’s response to stress. However, there are few reports about this gene family in peppers (*Capsicum annuum* L.). In this study, the pepper inbred line “Zunla-1” was used as the reference genome, and a total of 43 *PHD* genes were identified, and systematic analysis was performed to study the chromosomal location, evolutionary relationship, gene structure, domains, and upstream cis-regulatory elements of the *CaPHD* genes. The fewest *CaPHD* genes were located on chromosome 4, while the most were on chromosome 3. Genes with similar gene structures and domains were clustered together. Expression analysis showed that the expression of *CaPHD* genes was quite different in different tissues and in response to various stress treatments. The expression of *CaPHD17* was different in the early stage of flower bud development in the near-isogenic cytoplasmic male-sterile inbred and the maintainer inbred lines. It is speculated that this gene is involved in the development of male sterility in pepper. *CaPHD37* was significantly upregulated in leaves and roots after heat stress, and it is speculated that *CaPHD37* plays an important role in tolerating heat stress in pepper; in addition, *CaPHD9*, *CaPHD10*, *CaPHD11*, *CaPHD17*, *CaPHD19*, *CaPHD20*, and *CaPHD43* were not sensitive to abiotic stress or hormonal factors. This study will provide the basis for further research into the function of *CaPHD* genes in plant development and responses to abiotic stresses and hormones.

## 1. Introduction

Zinc-finger protein transcription factors are widely distributed among the eukaryotes. They are a type of protein with a “finger” domain and are rich in histidine and cysteine residues [1]. Based on the domain arrangement characteristics of the zinc-binding amino acids, zinc-finger proteins can be divided into different types, such as the plant homeodomain (PHD) motif (Cys_4_-His-Cys_3_), the really interesting new gene (RING) motif (Cys_3_-His-Cys_4_), and the LIM domain (Cys_2_-His-Cys_5_) [2,3]. The conserved motifs of PHD, RING, and LIM are very similar, but the spatial structures of the proteins are very different [4,5]. The PHD domain is a highly conserved motif (Cys_4_-His-Cys_3_) composed of about 60 amino acids [C-X2-CX (8–25)-CX (2–4)-CX (4–5)-HX2-CX (12–32)-CX (2–3)-C] [6].

With the rapid development of high-throughput sequencing technology, PHD family proteins are being identified and systematically studied in lots of plants. There are a large number of members of the *PHD* gene family in plants, with 67 in maize (*Zea mays* L.) [7], 73 in poplar (*Populus trichocarpa*) [8], 59 in rice (*Oryza sativa* L.) [9], 45 in tomato (*Solanum lycopersicum* L.) [10], and 106 in carrot (*Daucus carota* L.) having already been identified [11]. The PHD-finger motif is a common structural motif found in plants and fungi, which has been reported to bind to nucleosomes and to function as a nuclear protein-interaction domain [12]. The PHD zinc-finger family plays an important role in the growth and development of plants, with *Arabidopsis* PHD-domain ALFIN1-like proteins promoting seed germination [13] and the *germostatin resistance locus 1* (*GSR1*) gene, which encodes a PHD-finger protein, regulating seed germination and dormancy through the auxin-signaling pathway [14]. At the same time, PHDs can regulate *SOC1*/*FT* chromatin conformation to achieve the regulation of flowering [15]. The *MALE MEIOCYTE DEATH 1* (*MMD1*) gene in pepper (*Capsicum annuum* L.) encodes a PHD-type transcription factor that regulates the development of pollen and the tapetum. This gene is highly homologous to the *Arabidopsis MALE STERILITY1* (*MS1*) gene, and *MMD1* expression may occur during meiosis. An *MMD1* knockout mutation can cause the death of male meiotic cells [16]. *ZmMs7* in maize encodes a PHD-finger protein, which is specifically expressed in maize anthers, causing transcriptional activation and playing a key role in tapetum development and pollen outer wall formation [17]. The *ms4* mutant locus was finely mapped to a 216 kb region in the soybean genome, which contains 23 protein-coding genes. Among them, *Glyma.02G243200* encodes a protein orthologous with *MMD1* in *Arabidopsis*, which has a PHD domain that may be involved in the development of male sterility [18]. In addition, *MS4* in soybean (*Glycine max*) [18] and *TDR INTERACTING PROTEIN3* [19] and *PERSISTENT TAPETAL CELL1* [20] in rice also encode a PHD protein; they could regulate the formation of male sterility.

There are only a few reports that have shown that PHD proteins are involved in plant stress responses. For example, 15 of the 67 *PHD* family genes in maize responded to drought and high salt stress [7], whereas 9 of the *PHD* family genes in poplars showed differential expression under salt, drought, and cold stress conditions [8]. The genome-wide identification and characterization of the expression of the soybean *PHD* family genes showed that only six *PHD* family genes, *GmPHD1–6*, had been cloned and showed a response to drought stress. Among them, the expression of *GmPHD4* and *GmPHD5* was induced by cold stress [21], whereas that of *GmPHD2* and *GmPHD5* regulated the response of plants to salt stress [21,22]. In addition, studies have shown that PHD proteins can participate in DNA methylation, playing an important role in this phenomenon [23].

The pepper belongs to the Solanaceae family, which originates from Central and South America and is cultivated all over the world. The world’s pepper production exceeds 40 million tons (www.fao.org, (accessed on 15 November 2022)); the area of peppers planted annually in China is of the order of 1.5 to 2 million hectares, accounting for 8–10% of the total planted vegetable crop area. *PHD* genes have been identified in lots of plants, but the identification and functional analysis of *PHD* genes in pepper have not yet been studied. In this study, genes encoding PHD transcription factors were obtained by screening them from the pepper whole-genome database, and their sequence characteristics, chromosomal location, evolutionary relationship, protein conservation motifs, and gene structure were comprehensively analyzed to provide valuable information on the evolutionary mechanism of pepper *PHD* genes. By analyzing the expression of *PHD* family genes in different tissues at different developmental stages and under different stress treatments, the organ-specific expression and stress-responsive gene expression patterns of *CaPHD* genes were found, allowing us to preliminarily explore the biological functions of *PHD* family genes in pepper. The *CaPHD* genes identified in the current study could be the key genes involved in the flower development and stress response of pepper, and they will provide future insights for further research on the biological functions of *CaPHD* genes, as well as act as a theoretical reference for pepper molecular breeding in the future.

## 2. Materials and Methods

### 2.1. Identification of the PHD-Finger Family Genes in Pepper

The protein sequences of pepper “Zunla-1” (http://peppersequence.genomics.cn/) and *Arabidopsis thaliana* (https://www.arabidopsis.org/Blast/index.jsp, (accessed on 20 November 2022)) were obtained from the websites listed to construct the local protein database. Based on previous reports, the HMM model file (ID: PF00628) of the PHD proteins was downloaded from the Pfam database (http://pfam.xfam.org/, (accessed on 21 November 2022)), and the hmmsearch program of the HMMER software package was used to download proteins from the reference genome of “Zunla-1” and *Arabidopsis thaliana*. Possible PHD protein sequence IDs were screened from the protein database (E-value threshold < 1 × 10^−5^). Using the Perl script to extract the sequence information of each candidate protein, the sequence of the preliminary protein file obtained was compared with that from the Pfam database to identify the characteristic domain contained in the candidate sequence and to select the protein sequence with a complete PHD domain to be a member of the PHD family of this species.

The online tool ExPASy (https://web.expasy.org/protparam/, (accessed on 21 November 2022)) was used to analyze the isoelectric point, the number of amino acids, and the molecular weight of each *PHD* gene family protein, and it was used to align the protein sequence of members of the PHD family of “Zunla-1” with the *Arabidopsis* database (https://www.arabidopsis.org/Blast/index.jsp, (accessed on 21 November 2022)) to identify the pepper genes with the greatest homology to the *Arabidopsis PHD* genes.

### 2.2. Analyses of Chromosomal Location, Phylogenetic Relationship, Gene Structure, Functional Domains, and Conserved Motifs of the CaPHD Gene Family

The online software tool Gene Structure Display Server 2.0 (GSDS) (http://gsds.cbi.pku.edu.cn/) was used to analyze the structure of the pepper *PHD* family genes, while Multiple Em for Motif Elicitation (MEME) (http://meme-suite.org/, (accessed on 25 November 2022)) was used to analyze the corresponding PHD proteins. The online tool ExPASy (https://web.expasy.org/protparam/) was used to analyze the isoelectric point, and the conserved PHD motif had an output value set at 10. The Mapgene2chrom (MG2C) online tool (http://mg2c.iask.in/mg2c_v2.0/) was used to locate each gene on a particular chromosome, with MEGA X software being employed to construct a phylogenetic tree using the Neighbor-Joining method.

### 2.3. Identification of Cis-Acting Promoter Elements and Gene Expression Patterns

TBtools was applied to the “Zunla-1” genomic data to extract the 2000 bp promoter sequence upstream of each *CaPHD* gene, and the *cis*-regulatory elements in the promoter region were predicted with PlantCARE [24]. The expression data of the pepper inbred lines 6421, 9704A, and 9704B from the *PHD* gene family were obtained from PepperHub (http://www.hnivr.org/pepperhub/) and our previous research [25,26], and the gene expression heatmap was constructed using TBtools [24].

### 2.4. Plant Materials and Responses to Abiotic Stresses and Phytohormone Treatments

Using the expression data of the high-generation inbred line 6421 [25], the cytoplasmic male-sterile line 9704A, and the maintainer line 9704B to analyze the expression level of the *CaPHD* gene family during the different flower developmental periods and under different abiotic stresses. In order to ensure the accuracy and synchronicity of the data, floral buds were collected at different stages according to their size, and the expression level of each gene was calculated as the average FPKM of three biological replicates. Gene expression in 6421 was quantified in response to hormone treatments, including treatments with gibberellic acid (GA_3_) (2 μM), abscisic acid (ABA) (30 μM), SA (2 mM), or methyl jasmonate (JA) (10 μM), or in response to exposure to abiotic stresses, such as mannitol (400 mM, to simulate drought), salt treatment (200 mM NaCl), heat stress (42 °C), or cold stress (10 °C). Control plants were mock-treated with nutrient solution only. Leaf and root tissues were collected from both the treated and control plants at 0, 0.5, 1, 3, 6, 12, and 24 h post-treatment [25]. The flower development period was divided into three periods (S1, sporogenous celldivision stage; S2, uninucleate microspore stage; S3, mature pollen stage) in 9704A and 9074B based on the size of the flower: S1 (A1, B1 (2.0–3.0 mm)), S2 (A2, B2 (5.0–6.0 mm)), and S2 (A3, B3 (8.0–10.0 mm)) [26].

### 2.5. qRT-PCR Analysis

Total RNA was isolated from the different samples of 9704A and 9704B using the RNAiso Plus reagent (TaKaRa) and treated with RNase-free DNase I. Subsequently, 0.5 μg of RNA was used for first-strand cDNA synthesis using a HiScript II 1st Strand cDNA Synthesis kit (Vazyme, Nanjing, China). qRT-PCR was performed using the LightCycler 96 (Roche, Basel, Switzerland) with the SYBR Green Premix Ex Taq™ II quantitative PCR system (TaKaRa, Dalian, China), and the primers of the DEGs and reference gene are listed in Table S1. The experiment was repeated to obtain three biological replicates, and three technical repeats were carried out for each biological repeat. Briefly, after 95 °C for 10 min, the amplifications were carried out with 35 cycles at 95 °C for 15 s and 60 °C for 30 s. The relative expressions of target genes were calculated using the 2^−ΔΔCT^ method.

## 3. Results

### 3.1. Genome-Wide Identification and Chromosomal Location of CaPHD Gene Family in Pepper

From the HMM file of the PHD proteins, 60 candidate members of the *PHD* gene family were identified from the genome data of the pepper “Zunla-1”, and the *PHD* genes with incomplete sequences of the domain were removed via Pfam. In total, 43 *PHD* gene family members were ultimately identified from “Zunla-1” and numbered according to their chromosomal position and location on the chromosome (Table 1). The open reading frame size of the pepper *PHD* (*CaPHD*) gene family members was 405–7251 bp, with the number of amino acids encoded by the protein being in the range of 134–2416 and the isoelectric point being between 4.53 and 9.35 (Table 1).

**Figure 1:**
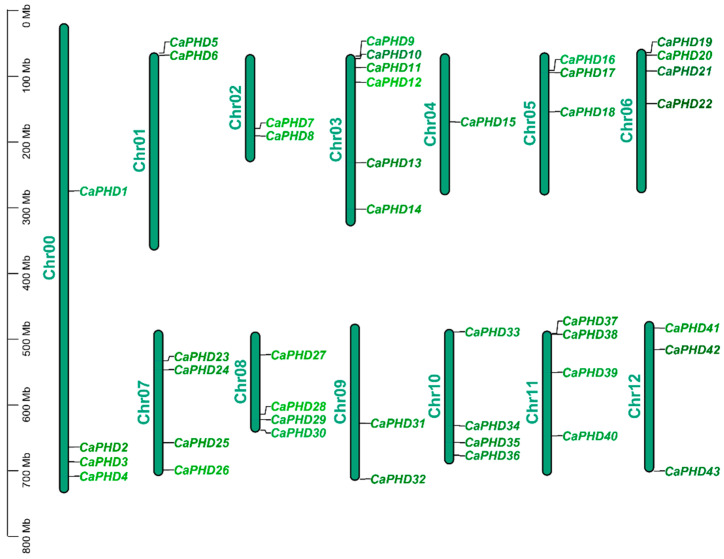
The chromosomal locations of *CaPHD* genes.

Based on the length of each “Zunla-1” chromosome and the distribution information of the *CaPHD* gene family members obtained from the genome, the MG2C online tool was used to draw the distribution map of the *CaPHD* genes on the 13 chromosomes. The 43 *CaPHD* genes were distributed across the “Zunla-1” chromosomes; chromosome 4 carried the fewest *CaPHD* genes (n = 1), whereas chromosome 3 carried the most genes (n = 5), with the remaining chromosomes, each containing between two and four *PHD* genes, and most of the *CaPHD* genes were located near the ends of chromosomes (Figure 1). The genome assembly proved to be incomplete, and four *CaPHD* genes that could not be mapped to any chromosome were placed on chromosome 0 (Figure 1).

### 3.2. Phylogenetic Analyses of the CaPHD Family

**Figure 2:**
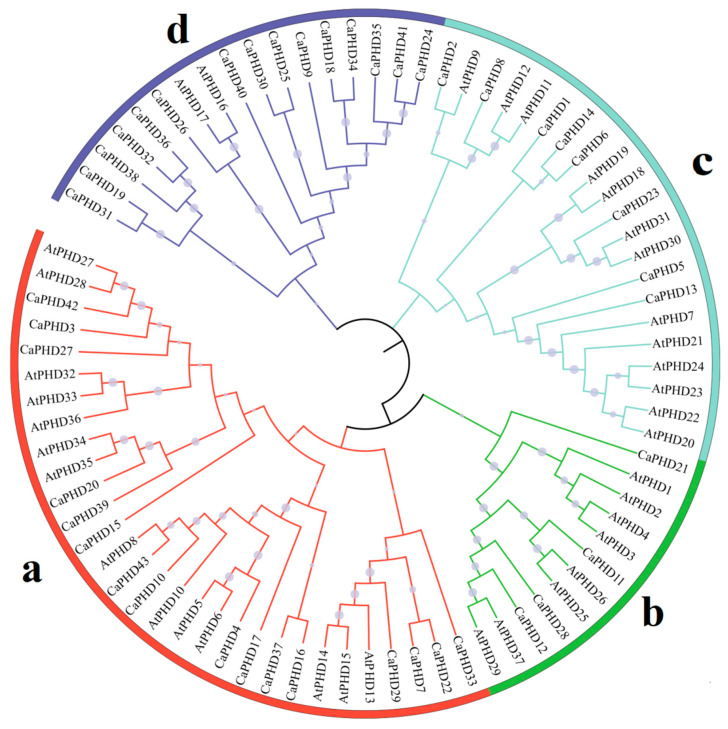
Phylogenetic analyses of PHD proteins from pepper (CaPHD) and Arabidopsis (AtPHD). The (a–d) stand for the subfamilies.

In order to better understand the evolutionary relationship between the *CaPHD* genes, we constructed a phylogenetic tree of the pepper *PHD* genes and their orthologs in *Arabidopsis*, and the results showed that the PHD proteins were clearly divided into four categories (Figure 2). Most of the *CaPHD* genes were located in the d category, whereas the *AtPHD* genes were located in the b and c categories. Furthermore, most of the subfamilies had a different number of genes between *Arabidopsis* and pepper.

### 3.3. Gene Structure and Conserved Motif Analysis of the CaPHD Family

**Figure 3:**
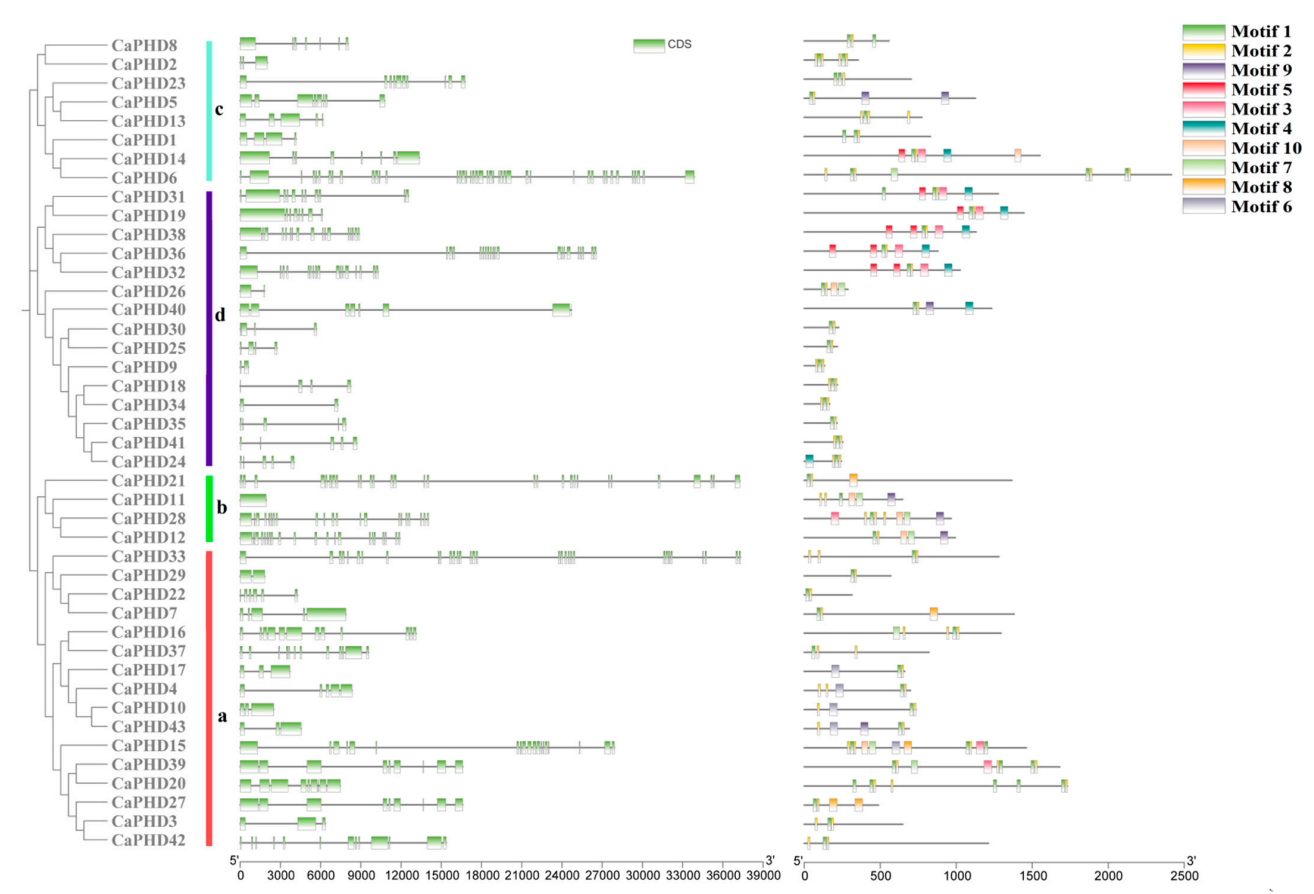
The structures of *CaPHD* genes and the conserved motifs of CaPHD proteins. The (a–d) stand for the subfamilies.

Determining the evolutionary relationship, structural analysis, and motif distribution of *CaPHD* gene family members can help us to better understand the similarity and diversity between the CaPHD protein family members. Cluster analysis showed that the 43 CaPHD members could be clearly divided into four groups, one of which (group b) contained only CaPHD11, CaPHD12, CaPHD21, and CaPHD28 (Figure 3). TBtools was used to map the structure of the pepper, and the results showed that most *CaPHDs* from the same subfamily shared similar gene structures. Conserved motifs of the CaPHD proteins were analyzed using MEME tools. All 43 CaPHD protein family members contained motif 1 and motif 2. Proteins CaPHD14, CaPHD22, CaPHD23, CaPHD25, CaPHD30, CaPHD34, and CaPHD41 each contained only motif 1 and motif 2, whereas at the other extreme, CaPHD15 contained 11 motifs. 

### 3.4. Analysis of Cis-Acting Regulatory Elements 

**Figure 4:**
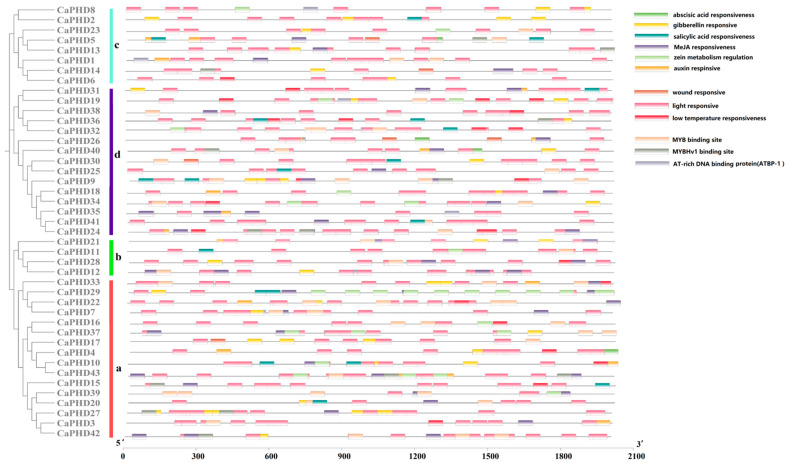
Predicted cis-regulatory elements in *CaPHD* promoters. Promoter sequences (2 kb upstream) of *CaPHD* genes were predicted using PlantCARE. The (a–d) stand for the subfamilies.

In order to study the response of the expression of the *CaPHD* genes to hormones and stresses, cis-acting regulatory elements were predicted and identified in the promoter region that is 2 kb upstream of the coding sequence of the *CaPHD* genes. The prediction results showed that the promoter region of the *CaPHD* gene family could respond to a variety of hormones (namely ABA, GA_3_, SA, or MeJA) and abiotic stresses (drought, low/high temperatures, or oxidative or salinity stresses) (Figure 4). In the results, 36 upstream cis-elements of the *CaPHD* genes were responsive to ABA, 32 to GA_3_, 32 to MeJA, 23 to IAA, and 15 to SA (Figure 4). In addition, a number of stress-responsive elements were identified, of which 17 were able to respond to low-temperature stress, whereas all 43 genes were responsive to light. In addition, the expression of *CaPHD* genes also occurred in response to zein metabolism, wounding, MYB, and AT-rich DNA-binding proteins (ATBP-1), among others. These results indicate that *CaPHD* genes may be related to various signal transduction pathways that involve phytohormones, light response, and abiotic stresses, suggesting that they may be involved in regulating the development and stress response of plants.

### 3.5. Tissue-Specific Expression Patterns of CaPHD Genes

**Figure 5:**
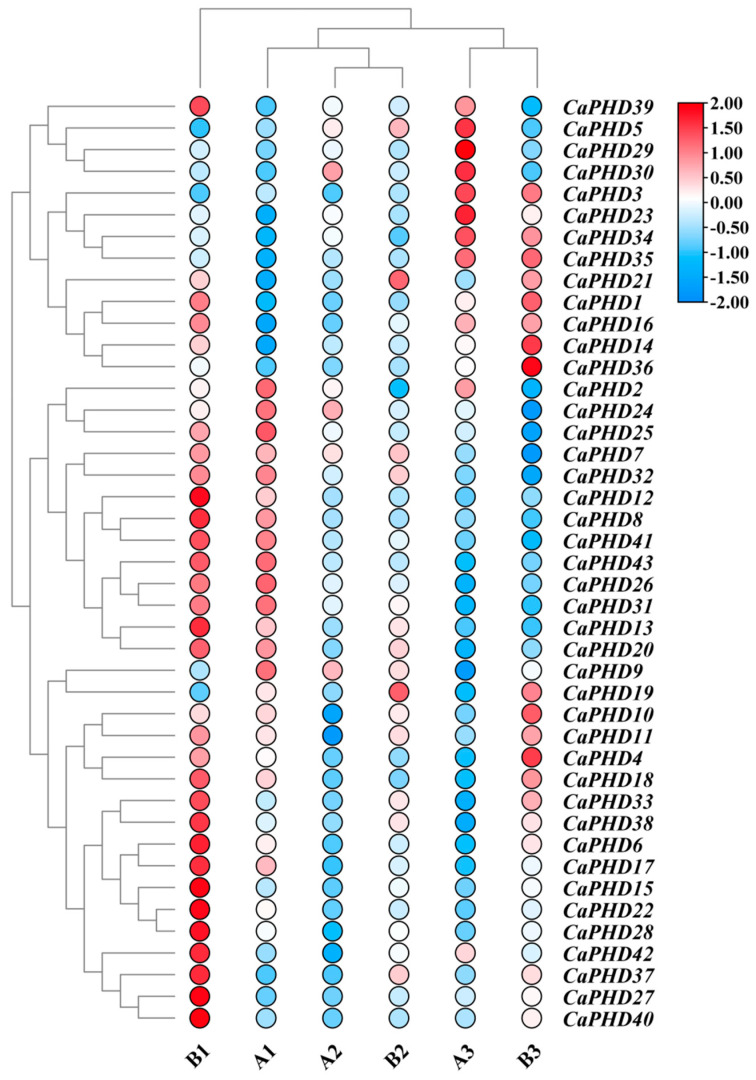
Expression patterns of *CaPHD* genes at different stages of flower bud development in 9704A and 9704B. A1–A3 and B1–B3 represent the sporogenous celldivision stage, uninucleate microspore stage, and mature pollen stage in 9704A and 9704B, respectively.

In order to predict the function of the *CaPHD* genes in the flower development in pepper, gene expression levels were analyzed at three different stages of flower bud development in the near-isogenic cytoplasmic male-sterile 9704A and maintainer 9704B lines (Figure 5, Table S2). The gene expression and cluster analyses in the male-sterile line 9704A and the male-fertile maintainer line 9704B show that A3, B3, A2, and B2 are clustered into one category, with A1 and B1 being clustered into a separate category. TheT expression levels were significantly higher in the B1 stage than in A1 for *CaPHD6*, *CaPHD15*, *CaPHD17*, *CaPHD22*, *CaPHD27*, *CaPHD28*, *CaPHD33*, *CaPHD37*, *CaPHD38*, *CaPHD40*, and *CaPHD42* (Figure 5). This indicates that the key period involved in flower development in pepper is the first stage. In addition, some differences between A3 and B3 were observed in the expression of several *CaPHD* genes. For example, the expression of *CaPHD10* and *CaPHD11* was significantly higher in B3. In total, 30 *CaPHD* genes exhibited obvious differences in their expression between the A1 and B1 period, being more highly expressed in the maintainer line than the male-sterile line. We speculate that *CaPHD6*, *CaPHD15*, *CaPHD17*, *CaPHD22*, *CaPHD27*, *CaPHD28*, *CaPHD33*, *CaPHD37*, *CaPHD38*, *CaPHD40*, and *CaPHD42* are mainly involved in the early stage of flower bud development, whereas *CaPHD4*, *CaPHD10*, *CaPHD14*, *CaPHD11*, and *CaPHD36* are predominantly involved in the late stage of flower bud development. These genes may also be involved in the formation of male sterility (Figure 5).

### 3.6. Expression Profiles of Stress-Related CaPHD Genes in Pepper

**Figure 6:**
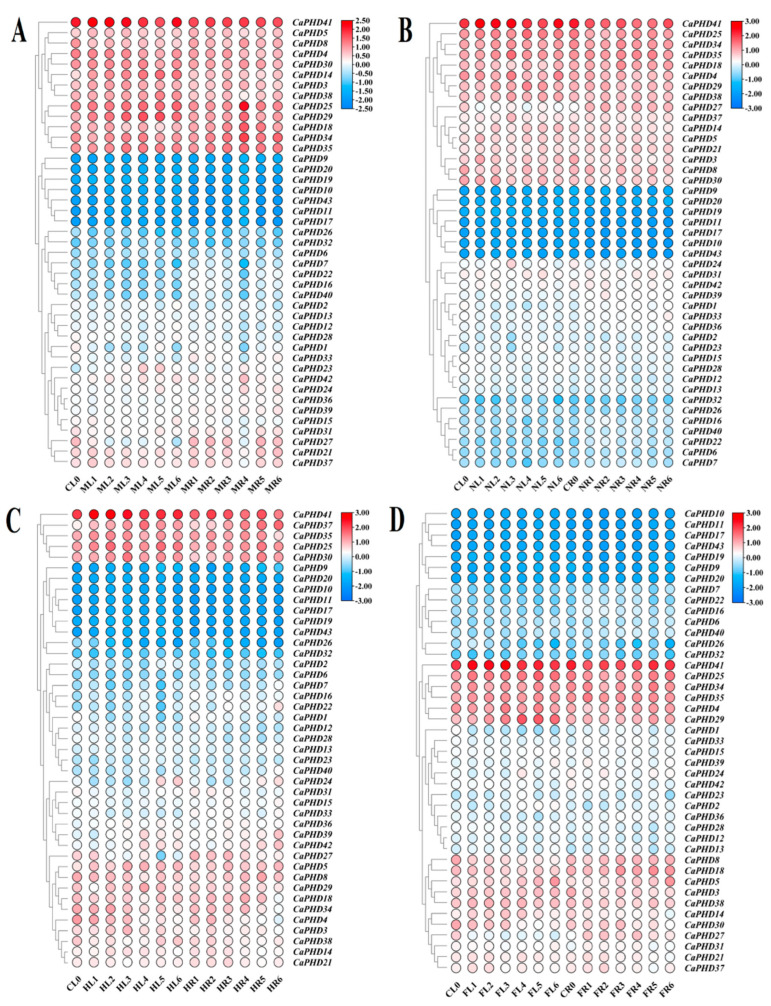
Expression heatmap of *CaPHD* genes under multiple abiotic stresses: (**A**) mannitol stress, (**B**) Nacl stress, (**C**) heat stress, (**D**) cold stress. The color scale represents log 2 (FPKM + 1) values. CL, ML, NL, HL, and FL represent the leaf samples treated with control, mannitol, Nacl, heat, and cold, respectively; CR, MR, NR, HR, and FR represent the root samples treated with control, mannitol, Nacl, heat, and cold, respectively; 0, 1, 2, 3, 4, 5, and 6 represent 0, 0.5, 1, 3, 6, 12, and 24 h post-treatment, respectively.

Previous studies have shown that the expression of *PHD* genes in other plant species can respond to drought, low temperatures, high temperatures, and salt stress by showing differences in expression under various stress treatments. We used the previous expression data to analyze the response of *CaPHD* genes in pepper leaves and roots to drought, low temperatures, high temperatures, and salt stress at different stages [25] and to identify *CaPHD* genes that could be involved in the regulation of the stress response (Figure 6, Table S3). Most of the *CaPHD* genes showed increased expression in roots and decreased expression in leaves under the simulated drought, low temperature, and salt stress, and this trend was most obvious in response to salt stress (Figure 6). For instance, *CaPHD25* and *CaPHD35* in the roots were both significantly upregulated under the four types of stress. The expression level of *CaPHD25* and *CaPHD34* was particularly sensitive to mannitol treatments, and changes in the expression of these two genes occurred mainly in the roots and reached the highest stage with 6 h treatment (Figure 6). The expression of *CaPHD29* occurred in response to cold stress, heat stress, and simulated drought stress in the leaves, showing a significant increase in expression under the 6 h treatment.

*CaPHD37* showed a unique response to heat stress, as it was upregulated by 7-fold after 6 h in the leaves and by 16-fold after 24 h in the roots. *CaPHD39* and *CaPHD42* were also involved in the response to heat stress in the roots (Figure 6). In addition, *CaPHD41* always maintained a high level of expression, which suggests that it is an essential gene in the tolerance to abiotic stress. *CaPHD9*, *CaPHD10*, *CaPHD11*, *CaPHD17*, *CaPHD19*, *CaPHD20*, and *CaPHD43* had a low expression and almost did not change after all of the stress treatments. We speculate that these genes are not involved in the response to drought, low temperatures, high temperatures, or salt stress. Comparing the four treatments, the *CaPHD* gene expression changed most obviously under high-temperature stress but was not sensitive to salt stress (Figure 6).

### 3.7. Expression Response of CaPHD Genes to Phytohormone Treatment

**Figure 7:**
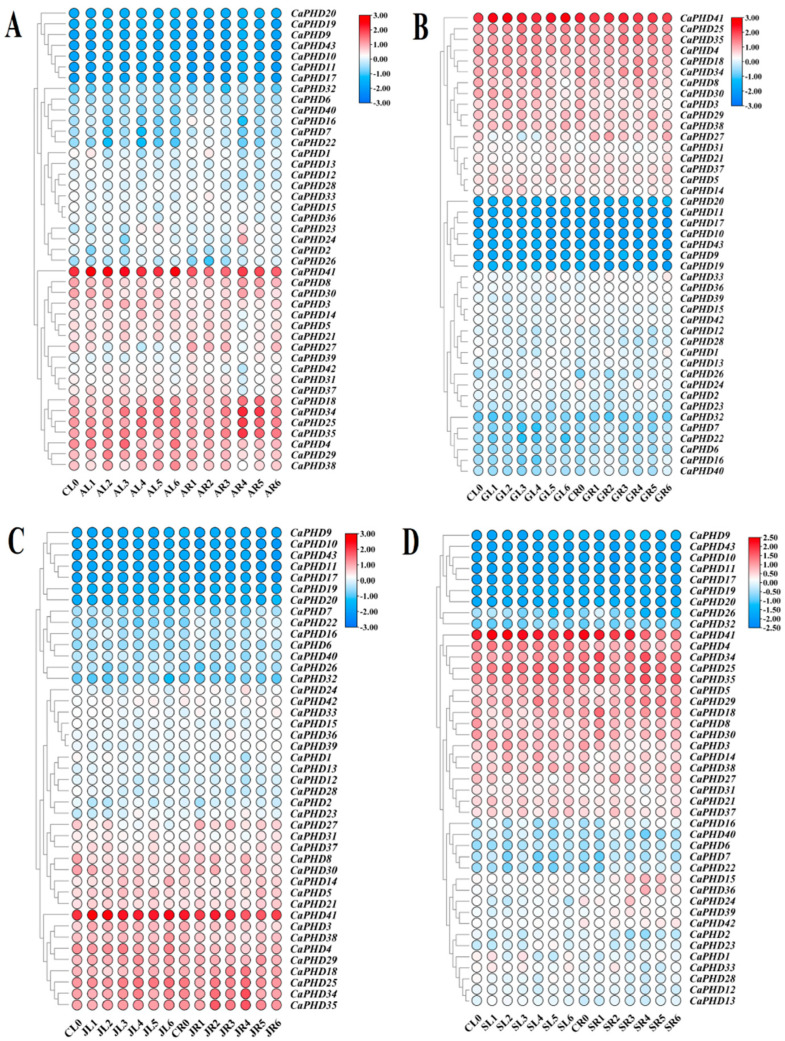
Expression heatmap of *CaPHD* genes in response to phytohormone treatment. (**A**) ABA stress, (**B**) GA3 stress, (**C**) JA stress, (**D**) SA stress. The color scale represents log 2 (FPKM + 1) values. CL, AL, GL, JL, and SL represent the leaf samples treated with control, ABA, GA3, JA, and SA, respectively; CR, AR, GR, JR, and SR represent the root samples treated with control, ABA, GA3, JA, and SA, respectively; 0, 1, 2, 3, 4, 5, and 6 represent 0, 0.5, 1, 3, 6, 12, and 24 h post-treatment, respectively.

We used previous expression data to analyze the expression pattern of *CaPHD* genes in pepper leaves and roots at six different time points (0.5 h, 1 h, 3 h, 6 h, 12 h, and 24 h) in response to a hormone treatment with ABA, JA, GA_3_, or SA (Figure 7, Table S4) [25]. The results showed that the expression of *CaPHD* genes responded similarly to the ABA, JA, GA_3_, or SA treatment. The *CaPHD* genes exhibited low levels of expression in the leaves and high levels of expression in the roots after hormone treatment. The response of *CaPHD* to JA was earlier than that to the other phytohormones, and it was expressed highest in the roots after 0.5 h of JA treatment (Figure 7). *CaPHD25*, *CaPHD34*, and *CaPHD35* exhibited the highest expression levels in roots in response to ABA, JA, GA_3_, and SA after 6 h of treatment, demonstrating that the above genes responded most significantly to hormone treatment after six hours (Figure 7). After treatment with any of the four hormones, the expression of *CaPHD21*, *CaPHD27*, *CaPHD31*, and *CaPHD37* in leaves showed a downregulated trend, whereas in roots, the expression was upregulated. *CaPHD41* always maintained a high level of expression, suggesting that this gene may play an important role in hormonal processing. *CaPHD9*, *CaPHD10*, *CaPHD11*, *CaPHD17*, *CaPHD19*, *CaPHD20*, and *CaPHD43* were also found to have almost not changed with phytohormone treatment; we speculate that these genes are not involved in response to the ABA, JA, GA_3_, and SA treatment (Figure 7).

### 3.8. qRT-PCR Validation of Gene Expression Patterns

**Figure 8:**
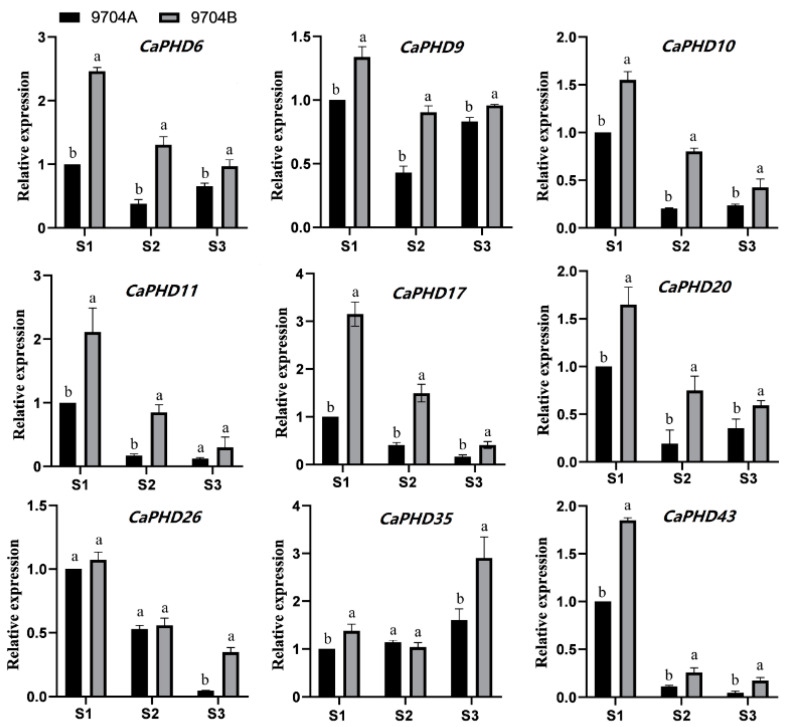
Nine *CaPHD* genes were selected to verify the expression level in 9704A and 9704B by qRT-PCR. S1–S3 represent the sporogenous celldivision stage, uninucleate microspore stage, and mature pollen stage in 9704A and 9704B; The significant differences test was performed using Duncan's test (*p* < 0.05), with different lowercase letters (a,b) indicating significant differences in mean values between different materials at the same stage.

In order to validate the results of RNA-Seq, nine *CaPHD* genes (*CaPHD6*, *CaPHD9*, *CaPHD10*, *CaPHD11*, *CaPHD17*, *CaPHD20*, *CaPHD26*, *CaPHD35*, and *CaPHD43*) were selected for further qRT-PCR analysis in different flower development stages (S1, sporogenous celldivision stage; S2, uninucleate microspore stage; S3, mature pollen stage) of 9704A and 9704B (Figure 8). These nine *CaPHD* genes showed the same tendency between the RNA-Seq analysis and qRT-PCR results, in which these genes were expressed higher in the first development stage of the male-fertile maintainer line 9704B, indicating that our transcriptome data were accurate and reliable.

## 4. Discussion

### 4.1. Identification and Phylogenetic Relationships of the CaPHD Gene Family in Pepper

Using the “Zunla-1” genome as the reference genome, a total of 43 PHD proteins were identified from pepper; this number was lower than that identified from maize (67), poplar (73), carrot (106), and cotton (*Gossypium hirsutum*) (108), but similar to the number identified from tomato (45). We speculate that this is due to the pepper and tomato both belonging to the Solanaceae family and thus having high levels of genomic similarity. According to the amino acid sequences encoded by the *CaPHD* genes, we carried out an evolutionary analysis of the 43 CaPHD proteins, where CaPHD was divided into four groups. The PHD proteins were categorized into eight groups in rice [9] and eleven or ten groups in maize [7,8]. Analysis of the structure of the *CaPHD* genes in pepper showed that the 43 *CaPHD* genes exhibited big differences in structure, which was similarly found in maize and rice [7,9]. Compared with previous studies on the evolution of PHD proteins in other species, our findings indicate that PHD proteins are more conserved in pepper than in other plants. The identification of motifs showed that two of the motifs were highly conserved as they were present in all 43 CaPHD proteins, suggesting that these two motifs may be associated with important functions of the PHD protein family.

Chromosomal localization indicated that most *CaPHD* genes were distributed in clusters, and most of them were located at the ends of the chromosomes. The distribution of *CaPHDs* may reflect a strategy for reducing variation and evolutionary divergence. Due to the large genome data on pepper, the diversity in gene structure is known to be closely related to the location of distribution and the range of functions it performs, and it can also predict the rate of evolution of the gene. Therefore, the distribution of *PHD* genes may provide a functional and structural framework for their role in reducing variation and evolution. Gene expression analysis revealed that *CaPHD9*, *CaPHD10*, *CaPHD11*, *CaPHD17*, *CaPHD19*, *CaPHD20*, *CaPHD43*, and other *CaPHD* genes exhibited minimal levels of expression in both the control plants and those exposed to various abiotic stresses. The coding regions of these *CaPHD* genes are shorter, and the lengths of amino acids are also small; these findings may be related to the evolution of the *CaPHD* genes in pepper.

### 4.2. CaPHDs Play Roles in Response to Developmental and Abiotic Stress

It has been reported that the *PHD* genes in plants are involved in the regulation of seed dormancy and in their subsequent germination [14], as well as being involved in the regulation of flowering [15]. In addition, *PHD* genes have been found to be widely involved in regulating the development of anthers and are closely related to the development of male sterility [16,17,18]. In the present study, most of the *CaPHD* genes were expressed differently between 9704A and 9704B in the early stage of flower buds. *CaPHD6*, *CaPHD15*, *CaPHD17*, *CaPHD22*, *CaPHD27*, *CaPHD28*, *CaPHD33*, *CaPHD37*, *CaPHD38*, *CaPHD40*, and *CaPHD42* may be involved in the early development of microspores, whereas *CaPHD4*, *CaPHD10*, *CaPHD14*, *CaPHD11*, and *CaPHD36* may be involved in regulating the fertility of pollen during the microspore maturation phase [26,27,28]. Homology studies have shown that *CaPHD17* is highly homologous to *MS1* in rice, with the latter regulating tapetal programmed cell death and pollen exine formation through its PHD finger [16,29,30]. We speculate that other *CaPHD* genes in the same group as *CaPHD17* may also be involved in the development of male sterility. *CaPHD10*, *CaPHD11*, and *CaPHD17* appeared to be involved in the development of flower buds, a phenomenon that may be functionally related to male sterility, but the expression of these genes was not sensitive to stress treatments. We speculate that the different expressions of *CaPHD* genes are significant in the early stage of bud development, and it may be the key point involved in the formation of male sterility.

*CaPHD9*, *CaPHD10*, *CaPHD11*, *CaPHD17*, *CaPHD19*, *CaPHD20*, and *CaPHD43* responded negligibly to high-temperature, low-temperature, simulated drought, salt, and hormone treatments. Among these genes, *CaPHD10*, *CaPHD11*, and *CaPHD17* are presumed to be involved in the development of flower buds, which could be closely associated with the development of male sterility. *CaPHD25*, *CaPHD34*, and CaPHD35 positively responded to ABA, JA, GA_3_, and SA phytohormone treatments. The expression of *CaPHD21*, *CaPHD27*, *CaPHD31*, and *CaPHD37* in leaves following treatment with any of the four hormones was downregulated. These findings are the first to report the response of *CaPHD* gene expression to ABA, JA, GA_3_, or SA treatment, providing a foundation for the relationship between *CaPHD* genes in response to treatment with the above four hormones, although the specific function of the *CaPHD* gene family needs further research and confirmation.

Previous studies on the *PHD* gene family have focused mainly on their role in development, but *PHD* genes can also participate in plant stress responses, although this involvement is supported by only a few reports. One study showed that the expression of 15 *PHD* family genes in maize could respond to drought or high salt stress [7], whereas another study determined that nine *PHD* family genes in poplars showed differential expression under salt, drought, or cold stress [8], and other studies on soybeans also showed that *PHD* family genes could respond to drought or salt stress [21,22]. The expression of *PHD* genes in upland cotton occurred in response to a range of abiotic stresses (and phytohormonal treatments) [31]. However, there have been few reports on the response of *CaPHD* family genes to abiotic stresses and phytohormonal treatments in pepper. The present study focuses on the response of *CaPHD* genes to high temperatures, low temperatures, simulated drought, and high salt levels. *CaPHD* genes showed differential expression in different tissues and developmental stages, indicating that they have specific expression characteristics that may relate to the specific functions of individual gene family members.

## 5. Conclusions

In this present study, a total of 43 CaPHD proteins were identified in pepper, with these CaPHD proteins being divided into four categories. Different *CaPHD* genes showed different expression patterns in response to different stress or hormone treatments and in different tissues, with strong spatiotemporal specificity. The expression analysis results from different developmental stages of flower buds suggest that some *CaPHD* genes may be involved in the development of flower buds and may be functionally related to the development of male sterility, a phenomenon that may be of significance to the generation of F_1_ hybrid cultivars. The expression analysis results of *CaPHD* genes in response to abiotic stress and hormone exposure also show that most *CaPHD* genes are involved in the regulation of stress tolerance, whereas a few genes are involved in only development but not in response to stress. Our research has provided a basis for identifying *CaPHD* gene family members and for screening for key genes with respect to particular functions while also providing a foundation for elucidating the functional mechanism by which *CaPHD* genes are involved in hormone response, organ development and abiotic stress response.

## Figures and Tables

**Table 1:** Detailed information on the pepper *PHD* gene family members.

Name	Gene ID	5′ End	3′ End	Protein Size (aa)	ORF (bp)	pI
*CaPHD1*	Capana00g000483	254,466,020	254,470,192	830	2493	5.47
*CaPHD2*	Capana00g004445	644,022,103	644,024,135	355	1068	5.59
*CaPHD3*	Capana00g004699	666,200,574	666,206,931	648	1947	8.35
*CaPHD4*	Capana00g005010	688,470,993	688,479,334	699	2100	8.22
*CaPHD5*	Capana01g000006	177,703	188,477	1127	3384	8.7
*CaPHD6*	Capana01g000238	3,671,024	3,704,883	2416	7251	5.78
*CaPHD7*	Capana02g001155	112,144,118	112,151,993	1381	4146	8.34
*CaPHD8*	Capana02g001395	123,396,735	123,404,780	558	1677	5.23
*CaPHD9*	Capana03g000140	1,991,155	1,991,777	134	405	4.53
*CaPHD10*	Capana03g000419	5,842,921	5,845,422	735	2208	6.14
*CaPHD11*	Capana03g001155	19,520,907	19,522,850	647	1944	8.64
*CaPHD12*	Capana03g002025	41,777,984	41789894	994	2985	8.93
*CaPHD13*	Capana03g003071	164,384,287	164,390,463	774	2325	8.48
*CaPHD14*	Capana03g003707	235,051,566	235,064,929	1551	4656	5.5
*CaPHD15*	Capana04g001765	103,459,026	103,486,934	1461	4386	7.81
*CaPHD16*	Capana05g000721	26,527,365	26,540,480	1295	3888	8.29
*CaPHD17*	Capana05g000766	29,933,832	29,937,546	661	1986	8.11
*CaPHD18*	Capana05g001230	89,689,887	89,698,139	219	660	5.3
*CaPHD19*	Capana06g000344	4,800,170	4,806,304	1446	4341	5.51
*CaPHD20*	Capana06g000580	8,805,536	8,813,012	1732	5199	7.04
*CaPHD21*	Capana06g001447	32,753,142	32,790,414	1367	4104	5.53
*CaPHD22*	Capana06g002050	82,405,508	82,409,778	314	945	8.19
*CaPHD23*	Capana07g000572	46,259,688	46,276,459	704	2115	8.64
*CaPHD24*	Capana07g000653	59,977,020	59,981,053	245	738	5.29
*CaPHD25*	Capana07g001300	170,904,485	170,907,239	219	660	7.09
*CaPHD26*	Capana07g002056	212,461,334	212,463,160	288	867	5.13
*CaPHD27*	Capana08g000308	34,753,957	34,763,289	488	1467	8.96
*CaPHD28*	Capana08g001093	124,758,585	124,772,634	967	2904	8.41
*CaPHD29*	Capana08g001577	132,947,991	132,949,834	570	1713	6.6
*CaPHD30*	Capana08g002581	149,165,449	149,171,136	227	684	8.48
*CaPHD31*	Capana09g001374	150,627,608	150,640,165	1277	3834	6.69
*CaPHD32*	Capana09g002280	235,766,586	235,776,872	1026	3081	6.44
*CaPHD33*	Capana10g000226	3,810,195	3,847,501	1281	3846	6.22
*CaPHD34*	Capana10g001358	146,143,245	146,150,538	168	507	6.05
*CaPHD35*	Capana10g001653	171,574,802	171,582,681	216	651	4.98
*CaPHD36*	Capana10g001914	191,133,928	191,160,485	880	2643	9.35
*CaPHD37*	Capana11g000110	3,151,600	3,161,184	821	2466	4.78
*CaPHD38*	Capana11g000184	4,429,857	4,438,746	1131	3396	4.91
*CaPHD39*	Capana11g000907	62,802,424	62,819,020	1681	5046	7.92
*CaPHD40*	Capana11g001326	159,279,171	159,303,879	1234	3705	5.97
*CaPHD41*	Capana12g000480	9,701,874	9,710,579	254	765	5.07
*CaPHD42*	Capana12g001061	42,716,016	42,731,391	1212	3639	5.38
*CaPHD43*	Capana12g002774	227,622,114	227,626,663	691	2076	5.72

## Data Availability

PHD gene members of pepper were downloaded from Pepperhub (http://pepperhub.hzau.edu.cn/Transcriptome/ExpCartoon.php, accessed on 3 May 2022). PHD gene members of A. thaliana were downloaded from the TAIR database (http://www.arabidopsis.org, accessed on 1 May 2022). The transcriptome datasets of 6421 were downloaded from Pepperhub (http://pepperhub.hzau.edu.cn/Transcriptome/ExpCartoon.php, accessed on 6 May 2022). The transcriptome datasets of 9704A and 9704B were deposited in the NCBI Short Read Archive (SRA, accession number: SRP186136) (accessed on 20 June 2022).

## Supplementary Materials

The following supporting information can be downloaded at https://www.mdpi.com/article/10.3390/genes14091737/s1, Table S1: The primers used for qRT–PCR in the study. Table S2. Expression patterns of *CaPHD* genes at different stages of flower bud development in 9704A and 9704B. Table S3: Expression of *CaPHD* genes under multiple abiotic stresses. Table S4: Expression of *CaPHD* genes in response to phytohormone treatment.
