# Supplementary material for: Characterization of Plant Homeodomain Transcription Factor Genes Involved in Flower Development and Multiple Abiotic Stress Response in Pepper
Source: Genes (Basel). 2023 Aug 30;14(9):1737. doi: 10.3390/genes14091737 (PMC10531376; doi:10.3390/genes14091737)
Supplement: Supplementary file 1 [file genes-14-01737-s001.zip › Table S1.pdf]

Table S1 The primers used in this study

Gene ID	Forward primer (5'-3')	Reverse primer (5'-3')
Capana05g000766	AATGGCTCATTGGAGATTGC	CCAAATTTGTAACCCCATCG
Capana08g001092	AGTCGACCGGTTTACATTGC	ACCGTGGATCCATCACTCTC
Capana07g000086	CAGGAAGCTTTCGGTAGCAC	CGAGCTTATCCTCTGGCAAC
Capana02g002161	TGGTGATGGCGTCAAGAATA	GTTGGTTGTGGGCGTAGAGT
Capana08g001455	AGCTCTCCGAACTCACGGTA	CTGATTCGTCACCACACCAC
Capana08g001555	ATGCTGCTGTTGTTGCAAAG	AAAGCGCAAGTGACACACTG
Capana09g000548	AAGCATGGTGAAGGGAATTG	CAACTGAGCAGCCATACGAG
Capana11g002314	GTGAAGGTCGTTGGAATGCT	TTTCGTTGTCCGTTCTTCCT
Capana08g000254	GGGACACCTTGCTAGAGCTG	GATGGTGATGCTGATTGTGG
β-Actin	TGCAGGAATCCACGAGACTAC	TACCACCACTGAGCACAATGTT
